# Assessing the Conformity of Mycelium Biocomposites for Ecological Insulation Solutions

**DOI:** 10.3390/ma17246111

**Published:** 2024-12-13

**Authors:** Ilze Irbe, Mikelis Kirpluks, Mikus Kampuss, Laura Andze, Ulla Milbreta, Inese Filipova

**Affiliations:** 1 Latvian State Institute of Wood Chemistry, Dzerbenes iela 27, LV 1006 Riga, Latvia; mikelis.kirpluks@kki.lv (M.K.); mikusskampuss25@gmail.com (M.K.); laura.andze@kki.lv (L.A.); ulla.milbreta@gmail.com (U.M.); inese.filipova@kki.lv (I.F.); 2 Faculty of Medicine and Life Sciences, University of Latvia, Raina bulvaris 19, LV 1586 Riga, Latvia; 3 Faculty of Natural Science and Technology, Riga Technical University, 6A Kipsalas iela, LV 1048 Riga, Latvia

**Keywords:** mycelium biocomposites, ecological insulation, *Trametes versicolor*, waste fibers, birch sawdust, hemp shives

## Abstract

In this study, different combinations of mycelium biocomposites (MBs) were developed using primary substrates sourced from the local agricultural, wood processing, and paper industries. The physicomechanical properties, thermal conductivity, and fire behavior were evaluated. The highest bending strength was achieved in composites containing waste fibers and birch sanding dust, with a strength competitive with that of synthetic polymers like EPS and XPS, as well as some commercial building materials. The lowest thermal conductivity was observed in hemp-based MB, with a lambda coefficient of 40 m·W·m^−1^·K^−1^, making these composites competitive with non-mycelium insulation materials, including synthetic polymers such as EPS and XPS. Additionally, MB exhibited superior fire resistance compared to various synthetic foams and composite materials. They showed lower peak heat release rates (134–243 k·W·m^−2^) and total smoke release (7–281 m^2^·m^−2^) than synthetic polymers, and lower total heat release (6–62 k·W·m^−2^) compared to certain wood composites. Overall, the mechanical and thermal properties, along with the fire performance of MB, support their potential as a sustainable alternative to petroleum-based and traditional composite materials in the building industry.

## 1. Introduction

Conventional insulation materials, such as fiberglass, mineral wool, polystyrene, and polyurethane foam, have long been the solution for improving energy efficiency in buildings. These materials are designed to reduce heat transfer, enhance thermal comfort, and lower energy consumption [1,2]. However, while they are widely used due to their availability and insulating properties, conventional insulation products often come with environmental concerns [3]. Many are made from non-renewable resources, have high embodied energy [4] and can release harmful chemicals during manufacturing, installation, or disposal [5]. As demand for more sustainable building practices grows, the limitations of traditional insulation materials are becoming increasingly evident. More than 30% of the EU’s environmental footprint comes from buildings, making it the sector with the highest environmental impact. About one third of the Union’s material consumption goes to construction. The use of buildings accounts for 42% of the total energy consumption and 35% of greenhouse gas emissions [6].

Renewable insulation materials have a lower environmental impact compared to conventional options. Although insulation materials derived from non-mineral, non-fossil, and non-wooden sources still hold a relatively small market share, they offer significant potential to reduce the environmental footprint of construction ventures [7]. The main opportunities for the integration of bio-based insulation materials in the European construction sector involve the gradual improvement with supportive policies, growing thermal, acoustic, and environmental performance, long-term cost savings, and increasing educational initiatives and advertising [8].

Natural fibers are eco-friendly, renewable materials that offer numerous advantages over synthetic fibers. They possess beneficial properties such as low weight, cost-effectiveness, low density, high specific properties, and availability from renewable resources. Extensive research has been conducted on natural fibrous materials to enhance the thermal insulation, mechanical, and physical properties of composite materials, making them highly promising for building applications [2]. A notable example is lime–hemp concrete, or hempcrete, a sustainable construction material made from hemp shives and lime as a binder [9]. Another type of sustainable biomaterial is mycelium composites (MBs), which are made from organic fibers bonded together by fungal mycelium. MBs do not generate non-recyclable waste and help reduce existing organic waste by utilizing the natural growth of fungi and agricultural or forestry by-products [10,11]. In recent years, MB research has gained increasing relevance due to its low production costs, ecological benefits, and high artistic value [12]. MB production requires less fossil energy and offers environmental advantages compared to conventional insulation materials. In particular, MBs have a lower climate change impact than materials like extruded polystyrene, foam concrete, and rockwool, although they require more land use and water compared to the conventional materials [13]. Moreover, MBs are fully biodegradable and compostable [14], making them an ideal choice for reducing the environmental impact of construction.

MBs can be engineered with varying properties, depending on factors such as the fungal species and strains used, the substrate composition, its structure and particle sizes, as well as the incubation conditions [15]. The substrate influences the concentrations of polysaccharides, lipids, proteins, and chitin in fungal hyphae, which in turn affect the material’s morphology and mechanical properties. This variability allows the properties of MBs to be tailored for different applications [16]. It also allows each substrate composition to be considered unique and unexplored until detailed tests and measurements have been carried out. The properties of potential building materials are particularly important for comparing them with standardized and frequently used materials and predicting their real applicability.

This study aimed to evaluate the potential of MBs as insulation materials for future applications in the building industry, focusing on their mechanical, thermal, and fire properties. Agricultural and wood processing by-products, along with waste fibers from the paper industry, were selected as the primary substrates in various combinations for MB development, with the basidiomycete *Trametes versicolor* serving as the substrate-binding agent.

## 2. Materials and Methods

**Figure 1:**
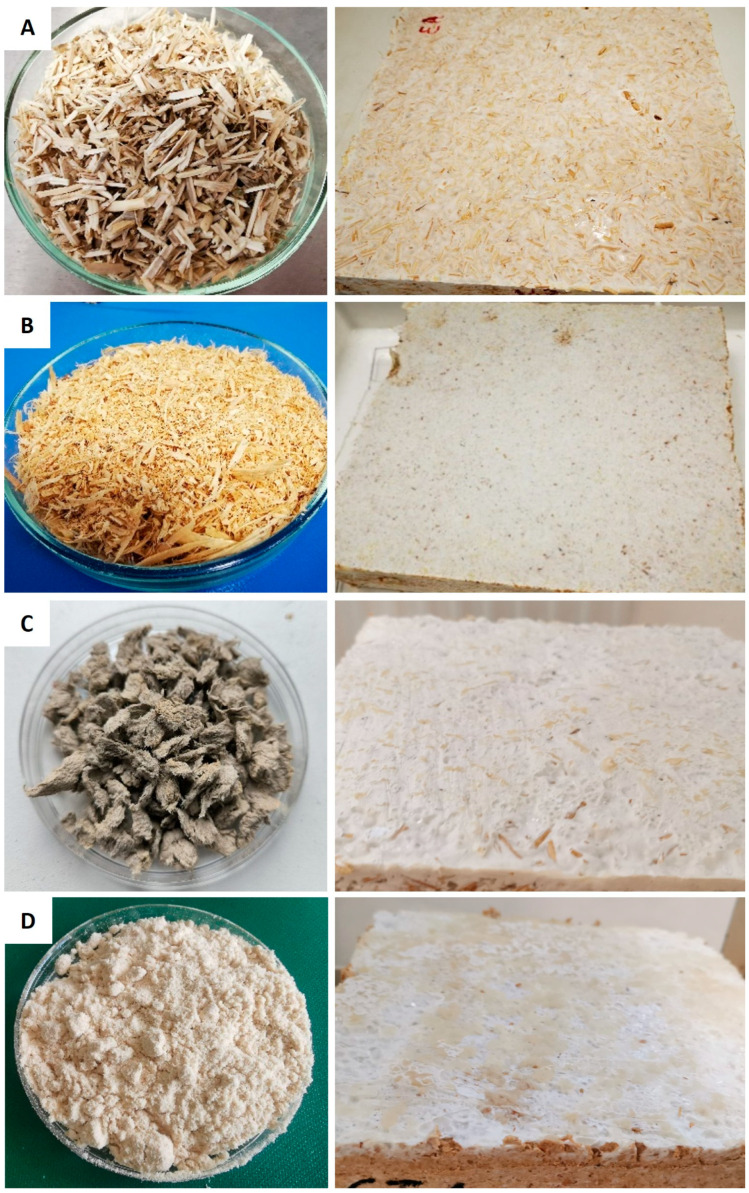
The main substrates and developed mycelium biocomposites with hemp shives (**A**), birch sawdust (**B**), waste fibers (**C**), and birch sanding dust (**D**).

The primary substrates and additives used to develop MBs were sourced locally. Hemp (*Cannabis sativa* L.) shives (HS) were obtained from agricultural waste, while silver birch (*Betula pendula* Roth) sawdust (BS) came from the wood processing industry. Birch sanding dust (BSD) substrate and birch bark (BB) additive were supplied by the plywood manufacturer AS “Latvijas Finieris”. Waste fibers (WF) were sourced from industrially recycled pulp provided by SIA “V.L.T.”, a molded fiber products company. Wheat bran (WB) additive was purchased from a retail supplier. The WF consisted of 60% mixed waste paper (such as newspapers, journals, books, office paper, and packaging paper), 30% cardboard, 5% printing house waste, and 5% waste from egg packaging production [17] (Figure 1). As noted in a previous study [14], the BB additive functioned as a potential water-repellent to enhance the material’s hydrophobic properties, while WB was used as a nutritional supplement.

Three different sample variants were prepared for each substrate group (Table 1), with 75% distilled water added to each variant.

The particle size and fraction percentages in the substrates were determined using an AS200 analytical sieve shaker (Retsch GmbH, Haan, Germany). The sieves were arranged vertically, with opening sizes of 10 mm, 7 mm, 5 mm, 3 mm, and 1 mm. After sieving, each granulometric fraction retained on the sieve was weighed, and its percentage relative to the total weight was calculated.

The fungus *Trametes versicolor* (L.) strain Quélet (FPRL 40C) was cultivated on MEA (5% malt extract, 3% agar) solid medium for 14 days at 22 ± 2 °C temperature and relative humidity (RH) 70 ± 5%, then used as inoculate for liquid medium. The mycelium was cultivated in a liquid medium containing 15.0 g glucose, 3.0 g peptone, 3.0 yeast extract, 0.8 g NaH_2_PO_4_, 0.4 g K_2_HPO_4_, 0.5 g MgSO_4_ on L water, pH 6, (all chemicals from Sigma Aldrich, Schnelldorf, Germany) in rotating shaker Multitron (Infors HT, Basel, Switzerland) for 14 days at 27 °C/150 rpm. After cultivation, mycelial pellets were homogenized by a Waring laboratory blender and mixed with sterile substrates in 4 L filter patch bags. Inoculated bags were incubated at 20 ± 2 °C and 70 ± 5% RH in the dark for 14 days. Afterward, the substrate-mycelium mixture from bags was transferred to molds with sizes 200 × 200 × 40 mm for the second growth phase. The molds were incubated in the conditions mentioned above for 7 more days, after which the developed MB samples were unmolded and dried at 70 °C for 24 h to inactivate fungal growth and calculate the moisture content. The final moisture content (%) of developed MBs was calculated from the wet and final dry weights. The MB plates were cut with a bandsaw into appropriate-size specimens for further analysis.

Micromorphology of the MB specimens was examined using a Leica S9i digital stereo microscope with a 10 MP integrated camera (Leica Microsystems, Wetzlar, Germany). The outer surface and cross-sections of the specimens were observed at magnifications ranging from 20× to 50×. Images were analyzed using Leica LAS V 4.12.0 software and stored in a PC database. For Scanning Electron Microscopy (SEM) 2 × 2 mm cubes located 1 cm from the sample surface were cut out and observed under SEM Tescan Vega TX (Brno, Czech Republic) to evaluate fungal distribution in the deeper levels of the samples. Before imaging, the samples were coated with gold plasma using a K550X sputter coater (Emitech, Chelmsford, UK). The samples were placed on graphite tape and scanned at a magnification 1000×.

MB specimens were weighed and manually measured in size in all three dimensions: length, width, and thickness, and with a digital caliper Scala Inox (Scala Messzeuge GmbH, Dettingen unter Teck, Germany). Specimen volume was calculated using Equation (1) and material density with Equation (2):

(1) $$V=\frac{l\times w\times h}{1000}$$

where: V = volume [cm^3^]; l = length [mm]; w = width [mm]; h = height [mm].

(2) $$\rho =\frac{m}{V}$$

where: ρ = density [g·cm^−3^]; m = mass [g]; V = volume [cm^3^].

Bending strength was detected for five parallel specimens with dimensions of 170 × 30 × 30 mm for each composition using the universal testing device Z010 (ZwickRoell, Ulm, Germany). The bending test procedure was carried out according to the standard EN 12089:2011 [18] in room conditions (23 ± 2 °C, RH 50 ± 5%). The pressure was applied at a speed of 10 mm min^−1^, and the pre-load was 1 N. Bending tests were performed in a three-point bending setup with a 150 mm span between supports. The maximal bending strength is calculated using Equation (3):

(3) $$\sigma_{fM}=\frac{3F_{m}L}{2bh^{2}}$$

where: *σ*_fM_ = the maximal bending strength [MPa]; F_m_ = the maximal applied force [N]; L = the span length [mm]; b = the width of sample [mm]; h = the thickness of sample [mm].

The modulus of elasticity is calculated using Equation (4):

(4) $$E=\frac{L^{3}}{4bd^{3}}\times \frac{F_{t}}{x_{t}}\times 10^{3}$$

where: E = modulus of elasticity [MPa]; L = the span length [mm]; b = the width of the sample [mm]; h = the thickness of sample [mm]; F_t_ = the force, corresponding to the deflection *x*_t_ [N]; *x*_t_ = the corresponding deflection [mm].

Thermal conductivity was measured using a Linseis HFM 200 in accordance with ISO 8301:1991 [19], with a temperature difference (ΔT) of 20 °C (cold plate: 0 °C, hot plate: +20 °C). Three replicate samples, each 200 × 200 × 30 mm, were tested.

**Figure 2:**
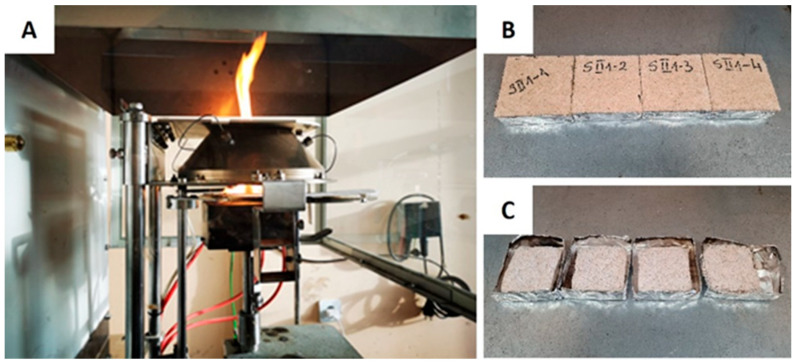
Cone calorimeter test procedure (**A**) and mycelium biocomposite with birch sanding dust substrate before (**B**) and after (**C**) burning.

The cone calorimetry method based on oxygen consumption is commonly employed to assess the heat release behavior during a fire. The reaction to fire was evaluated using an FTT Dual Cone Calorimeter in accordance with ISO 5660-1:2015 [20] MB specimens, measuring 100 × 100 × 30 mm, were subjected to a constant heat flux of 35 k·W·m^−2^ (Figure 2).

The statistical analysis was conducted using RStudio (R version 4.2.1). Differences with a *p*-value < 0.05 were considered significant. One-way ANOVA was employed to assess the significance of differences across groups, followed by Tukey’s HSD post hoc tests for pairwise comparisons. The following R packages were utilized: agricolae for performing the ANOVA and Tukey HSD tests, dplyr for data manipulation, readxl for reading Excel files, and writexl for exporting results (Supplementary Materials).

## 3. Results and Discussion

The role of substrates in mycelium composites is crucial, as the substrate serves as the primary growth medium for the mycelium and greatly influences the final properties of the composite material. Different substrates lead to varying concentrations of polysaccharides, proteins, lipids, and chitin in the mycelium, which affects the morphology, density, and overall performance of the composite. This allows for tailoring the mycelium composite to specific applications by altering the substrate [16].

### 3.1. Granulometry

**Figure 3:**
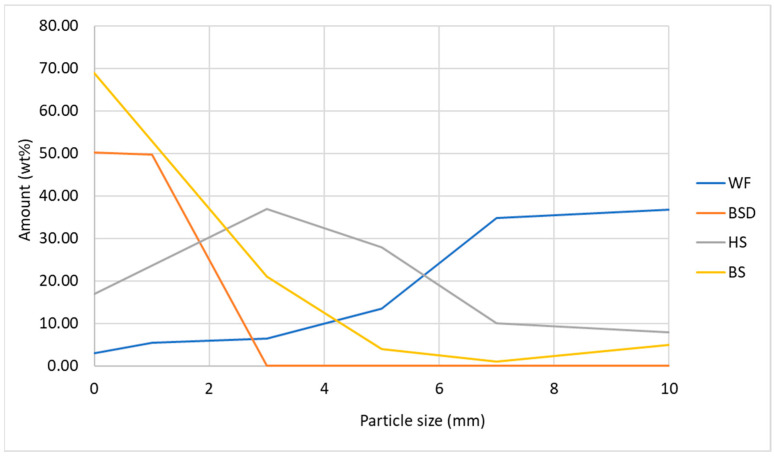
Particle size distribution of the substrates. HS = hemp shives; BS = birch sawdust; WF = waste fibers; BSD = birch sanding dust.

Particle size distribution was strongly dependent on the substrate and affected the MB density. In hemp substrate, 3–5 mm particles prevailed over other sizes (Figure 3).

BS primarily consisted of particles measuring ≤3 mm, BSD was limited to ≤1 mm. It is important to note that the dry WF, when sieved, tended to clump together, forming larger aggregates with particle sizes of 7–10 mm (Figure 1C). During the composite preparation stage, wetting caused these aggregates to break apart into a homogeneous mass of smaller fibers.

### 3.2. Micromorphology

MBs result from a biological process, making adequate moisture in the substrate essential for fungal development. In the finalized MBs, the average moisture content was 57% for the hemp (C) group, 50% for the sawdust (D) group, 64% for the waste fibers (WF) group, and 67% for the sanding dust (S) group. (substrate groups are defined in Table 1).

**Figure 4:**
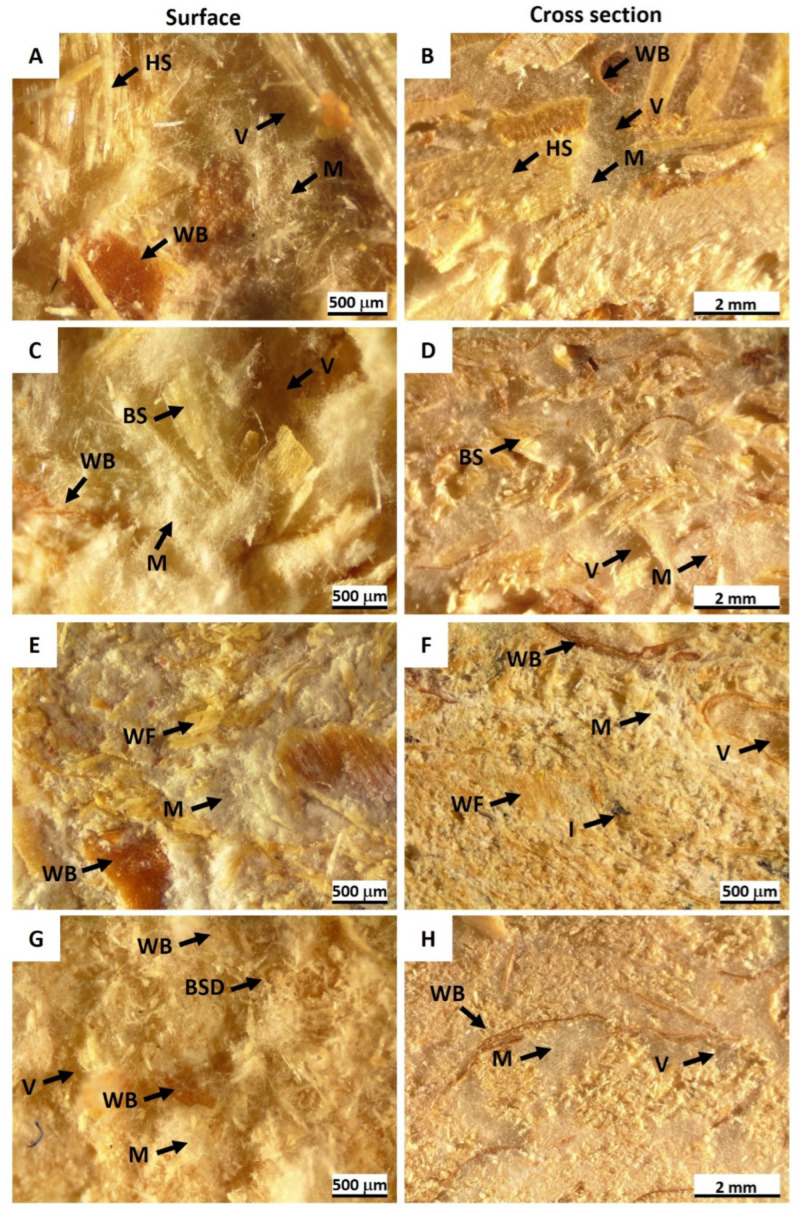
Microstructure of mycelium biocomposites derived from *Trametes versicolor* mycelium and different substrates: (**A**,**B**) hemp (HS)/wheat bran (WB); (**C**,**D**) birch sawdust (BS)/WB; (**E**,**F**) waste fibers (WF)/WB; (**G**,**H**) birch sanding dust (BSD)/WB. Arrows indicate substrates with WB, mycelium (M), voids (V), and ink (I). Scale bars 500 µm (**A**,**C**,**E**,**F**,**G**) and 2 mm (**B**,**D**,**F**).

The adequate moisture content of the substrates promoted fungal growth both on the outer surface and within the cross-section of the developed MB variants. The hemp composites (C1 variant) exhibited a heterogeneous particle distribution with a porous structure, where the voids were filled with sparse fungal mycelium. Hyphae were also observed attaching to the hemp and wheat bran (WB) particles (Figure 4A,B). In contrast, the sawdust samples (D1 variant) displayed a more compact particle arrangement, particularly in the inner layers, with fewer voids and thicker mycelial colonies (Figure 4C,D). This observation aligns with the granulometric analyses, which showed that particle sizes of ≤3 mm predominated in the sawdust substrate (Figure 3). Dense mycelium was present both on the surface and within the cross-section of the sawdust composites.

Waste fiber (M variant) samples exhibited a distinct morphological pattern compared to the hemp and sawdust composites, characterized by a notably compact and dense arrangement of substrate particles and mycelial colonies, with only a few dispersed voids. The presence of residual ink particles, which can be expected because of waste paper is used, did not appear to hinder hyphal growth (Figure 4E,F). Similarly, the sanding dust (S variant) substrate led to the formation of a homogeneous MB material, featuring a dense network of substrate and mycelium both on the surface and within the inner layers of the composite (Figure 4G,H).

**Figure 5:**
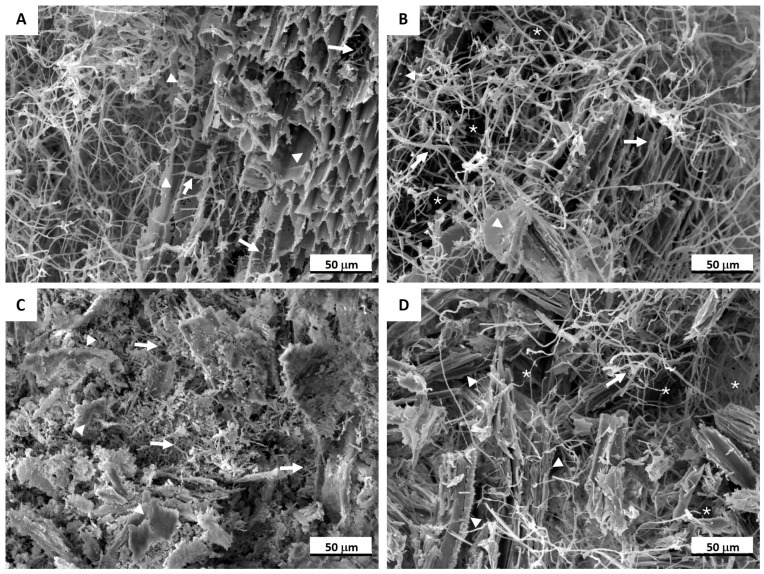
SEM images of mycelium biocomposites in cross-section: (**A**) hemp substrate (HS); (**B**) birch sawdust (BS) substrate; (**C**) waste fibers (WF) and (**D**) birch sanding dust (BSD) substrates. Arrows indicate the fungal hyphae and arrowheads mark the substrates; asterisks indicate voids. Scale bar 50 µm.

SEM micrographs illustrating the distribution of fungal hyphae within the MBs are presented in Figure 5. In the HS substrate (Figure 5A), sparse mycelium was observed filling the voids between substrate particles, with hyphae distributed along the woody fibers of the shives (arrows), utilizing them as a nutrient source. In the BS composites (Figure 5B), dense mycelium was observed filling the voids and connecting the substrate particles. Additionally, hyphal distribution was evident both along and between the individual wooden cells. The WF were covered by dense mycelium (Figure 5C), which also extended throughout the substrate particles. The BSD composites exhibited a compact microstructure (Figure 5D), characterized by small voids (asterisks) and a hyphal network enveloping the individual wood cell particles (arrows), resulting in a homogeneous composite structure.

The SEM micrographs confirmed that all substrates facilitated hyphal distribution by overgrowing the substrate particles and penetrating the structure of individual substrate fibers. Mycelial growth was also noted in the empty spaces of the composites, which acted as air pockets to support continuous hyphal growth.

### 3.3. Bending Strength

The physico-mechanical properties of the MB variants are presented in Table 2. ANOVA indicated significant differences in material density, bending strength, and elastic modulus (*p* < 0.001). The D3 variant was excluded from this analysis due to insufficient replicates, as its weak structure collapsed when pressure was applied. This can be attributed to the use of pure sawdust substrate without the addition of wheat bran (WB) as a nutritional supplement. WB is recognized as an effective growth substrate that promotes lignocellulolytic enzyme production in many basidiomycetes [21]. As a result, fungal growth was limited, leading to extremely low mechanical strength in the sawdust composite.

The density of the MB variants was influenced by both the primary substrate and the combinations with co-substrates and additives. The densities of the MB variants ranged from 0.09 to 0.32 g·cm^−3^. The lowest density was observed in the hemp-containing composites (C1), which had a density of 0.1 g·cm^−3^, followed by the sawdust (D1) and sanding dust (S1) groups, each with densities around 0.2 g·cm^−3^. The waste fiber samples (M1) exhibited the highest density, reaching 0.3 g·cm^−3^ (Table 2). According to post hoc analysis, significant differences were found among all groups, except between C3 and C1, S2 and C2, M3 and D2, S3 and D2, S1 and D1, and S3 and M3. Similar densities ranging from 0.10 to 0.17 g·cm^−3^ have been reported for mycelium composites made with rapeseed straw and beech sawdust [22]. In comparison, traditional construction or insulation materials have densities that vary from 0.02 g·cm^−3^ for synthetic foams like expanded polystyrene (EPS) and extruded polystyrene (XPS) [23,24] to 0.7 g·cm^−3^ for wood composite OSB [25].

There was a significant variation in bending strength, ranging from 0.10 to 0.51 MPa, and elastic modulus, ranging from 4.10 to 26.66 MPa, across all MB variants (Table 2). The hemp variant C2, which included a birch bark (BB) additive, exhibited the highest density and a bending strength of 0.15 MPa within the C substrate group. In the sawdust substrate group (D), the highest bending strength of 0.25 MPa and an elastic modulus of 14.66 MPa were recorded for variant D1.

Among all substrate groups, waste fibers (M1–3) demonstrated the highest bending strength, ranging from 0.39 to 0.45 MPa, with variant M1 showing an elastic modulus of 26.66 MPa, significantly differing from all other substrate groups. The higher elasticity in the material is likely due to the use of pure waste fibers derived from various wood fibers with an average length of 1.1 mm and an average width of 25 µm [17]. These natural fibers tend to have flexible and resilient properties, which can contribute to the overall elasticity of the MBs. Wood fibers, such as cellulose, are known for their strength and flexibility [26], which may enhance the mechanical performance of the composite, particularly in terms of elasticity and deformation resistance. The opposite results were reported by Teeraphantuvat et al. (2024) [27], where the addition of paper waste to sawdust containing mycelium composites did not improve their flexural strength which decreased from 0.090 MPa to 0.018 MPa when 40% paper waste was added.

A low strength within the sanding dust (S) group exhibited the S1 variant with σ_fM_ 0.10 MPa. In comparison, the highest value of σ_fM_ 0.51 MPa and elastic modulus 17.87 MPa showed the S2 variant with added hemp and WB supplement (Table 2). Comparing the S1, S2, and S3 variants with each other, the strength of the S1 variant was significantly lower than that of the S2 and S3 variants, while the strength of the S2 variant was significantly the highest. This variation can be attributed to the small particle size of the S1 substrate (≤1 mm) compared to S2, which had the added hemp co-substrate. Similarly, the sawdust co-substrate in the S3 variant increased the bending strength to 0.32 MPa.

Similar values of flexural strength were recorded for non-pressed *Trametes ochracea* composites containing beech sawdust (0.29 MPa) and rapeseed straw (0.22 MPa) substrates [22]. However, these results are lower compared to the bending test results obtained in this study, where the highest bending strength values of 0.51 MPa and 0.45 MPa were achieved using substrate combinations of sanding dust/hemp and waste fibers/sawdust, respectively.

Currently, mycelium-based materials cannot compete with the types of oriented strand board (OSB) composites, which have significantly higher flexural strengths ranging from 19 MPa [25] to 21–28 MPa [28]. In contrast, the bending strength of MB materials is competitive with synthetic polymers, such as expanded polystyrene (EPS) with 0.3 MPa [23] and extruded polystyrene (XPS) with bending strengths of 0.5 to 0.6 MPa [24]. Furthermore, a commercial hempcrete product exhibits a flexural strength of 0.30–0.40 MPa [9], which is comparable to that of MB materials.

### 3.4. Thermal Properties

**Figure 6:**
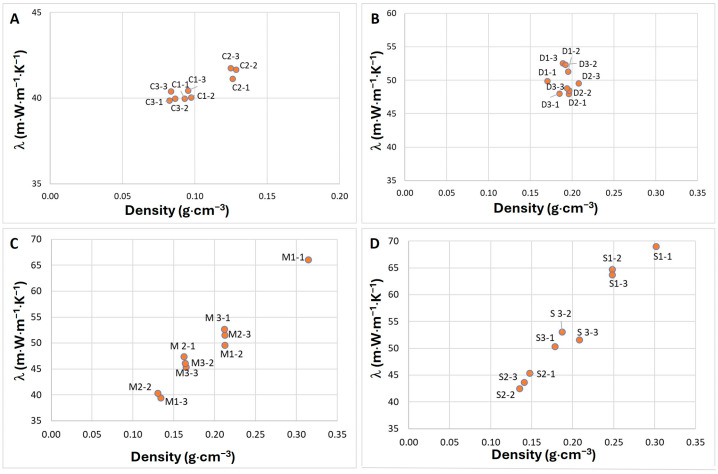
Thermal conductivity of mycelium composites based on hemp C (**A**), birch saw-dust D (**B**), waste fibers M (**C**), and birch sanding dust S (**D**) substrates; n = 3.

The thermal conductivity of the MB materials is shown in Figure 6. The ANOVA revealed significant differences in material density and thermal conductivity (*p* < 0.001).

Since the MB samples within each substrate group had varying densities, the lambda values were influenced and differed for each specific sample within the group.

The thermal conductivity of hemp C (Figure 6A) composites was lower, averaging between 40 to 41 m·W·m^−1^·K^−1^, compared to the other substrate groups (Figure 6). This lower thermal conductivity was attributed to the lower apparent density of the hemp-containing MBs, which reduced the lambda value. The thermal conductivity of birch sawdust D (Figure 6B) composites was around 50 m·W·m^−1^·K^−1^, with nearly twice higher density than hemp MBs. The thermal conductivity for waste fiber M (Figure 6C) composites ranged from 46 to 51 m·W·m^−1^·K^−1^. It was influenced by both the specific variant and the apparent density of individual samples. The average lambda values for the sanding dust S (Figure 6D) group were more varied, ranging from 44 to 64 m·W·m^−1^·K^−1^, depending on the sample’s apparent density. Adding hemp co-substrate in the S group significantly reduced apparent density and the average lambda coefficient to 44 m·W·m^−1^·K^−1^. Variant S1, which contained sanding dust and WB additive, exhibited the highest apparent density and lambda values, showing significant differences from all other groups.

Thermal conductivity previously reported for *Trametes versicolor* composites ranged from 54 m·W·m^−1^·K^−1^ [29] to 64 m·W·m^−1^·K^−1^ [30] on beech sawdust, which aligns with the values observed for birch substrates used in this study. The wooden particles contribute to higher apparent density and, consequently, higher thermal conductivity. Holt et al. (2012) [31] obtained mycelium composites with a thermal conductivity between 100 and 180 m·W·m^−1^·K^−1^ by inoculating different fractions of cotton agricultural residues, starch, and gypsum with *Ganoderma* sp., which is more than double the thermal conductivity results found in this study. Wimmers et al. (2019) [32] utilized five different tree species as substrates and nine fungal species as inoculum to determine which combinations of wood fibers and selected wood rot fungi were most suitable for panel production. This research resulted in boards with a similar thermal conductivity of around 50 m·W·m^−1^·K^−1^ for all panels, slightly higher than the lower thermal conductivity results obtained in this study. The lambda value of 40 m·W·m^−1^·K^−1^ observed for hemp MBs in this study indicates that hemp-based MB samples are more successful options for thermal insulation solutions.

The hemp-based MBs are also competitive with non-mycelium insulation materials in terms of thermal conductivity properties. The lambda coefficients of synthetic foam materials, such as EPS and XPS, are slightly lower than MBs, 37 and 38 m·W·m^−1^·K^−1^, respectively [33]. Low-density glass wool, rock wool, and wood fiber wool insulation materials have lambda coefficients of 36, 40, and 50 m·W·m^−1^·K^−1^, respectively [33]. Currently, rigid polyurethane foam (RPUF) is an insulation material with one of the lowest thermal conductivity of 18–24 m·W·m^−1^·K^−1^ available on the market [34], approximately half that of MBs.

However, RPUF has significant drawbacks. Its thermal conductivity tends to increase over the exploitation of the material due to the blowing agent gas diffusion from the material [35]. The gas diffusion is mitigated by inclosing the RPUF in gas tight barrier, such as metal sheet in sandwich type polyisocyanurate foam panels or by producing soft shin RPUF foam panels covered with aluminum foil. Most of the RPUF thermal insulation materials commonly used is still sourced from petrochemical feedstock that is not renewable [36]. Lastly, the RPUF releases toxic smoke during burning due to the inherent chemical structure of the material [37]. The thermal conductivity of MBs could potentially be improved by incorporating extra light ingredients, creating a more porous structure, and reducing density. The MB composites hold a significant advantage over thermal insulation materials because they are produced entirely from bio-based feedstock.

### 3.5. Reaction to Fire

**Figure 7:**
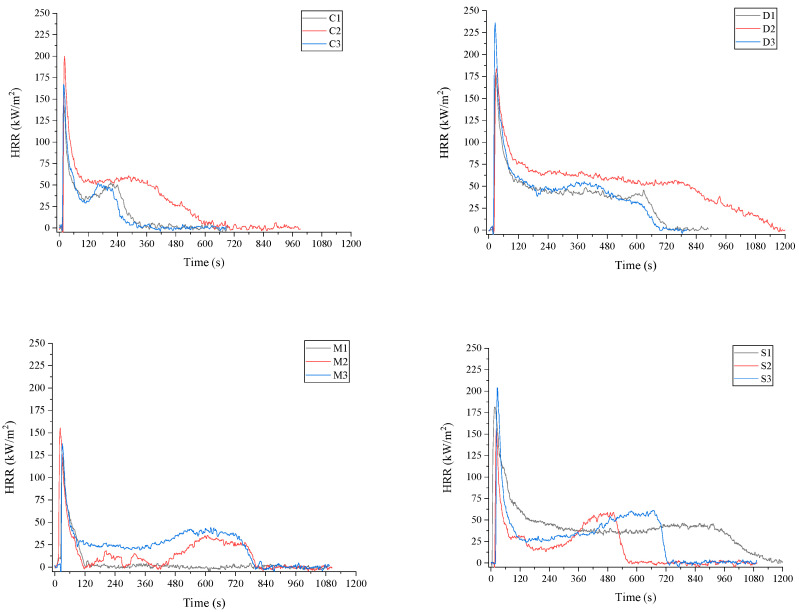
HRR curves of C (hemp), D (birch sawdust), M (waste fibers) and S (birch sanding dust) containing mycelium composites. The numbers 1, 2, 3 indicate the different variants of each substrate group.

Table 3 presents the cone calorimetry test results of the MB samples, including key fire performance metrics: time to ignition (TTI), time to flameout (TTF), total heat release (THR), peak heat release rate (pHRR), time to peak heat release rate (TTP), total smoke release (TSR), and the heat release rate (HRR) (Figure 7). For comparison, the fire characteristics of EPS and XPS [38] which are commonly used insulation materials in buildings, have also been included in Table 3. ANOVA indicated significant differences across all tested parameters, such as TTI, TTF, THR, pHRR, and TSR (*p* < 0.001).

Time to ignition (TTI) refers to the period between the start of heat exposure and the point when sustained flaming occurs. For the MB samples, TTI ranged from 9 to 18 s, while synthetic foams XPS ignited in 5 s, and EPS in 29 s (Table 3). The shorter ignition times for hemp composites (C group) and hemp-containing variants, such as M2 (waste fibers) and S2 (sanding dust), indicate a higher ignition risk compared to birch sawdust-containing MBs (D group and M3, S3). The ignition behavior was influenced by apparent density and surface roughness of the samples and thickness of the composites outer layer. For example, the hemp composites (C group), which had the lowest density (Figure 6A), showed the shortest ignition times. Similar TTI values have been reported for other MB materials, such as rice hulls and wheat grains, which ignited at 7 and 12 s, respectively [39]. However, all MB variants exhibited longer TTI than XPS (Table 3). Due to their higher density and composition, OSB and plywood demonstrated longer ignition times, ranging from 24 to 25 s [40,41].

MBs consist of organic matter, including fiber biopolymers such as polysaccharides, lignin, and fungal hyphae, which contain chitin, glucans, mannans, glycoproteins, and glycolipids. The thermal degradation of natural fibers occurs in stages: hemicellulose decomposes around 250–370 °C, α-cellulose breaks down between 340 and 370 °C via depolymerization, and lignin decomposes in the temperature range of 200–500 °C [42]. Fungal mycelium, begins decomposing at approximately 250 °C [39]. These factors mutually influence the flammability behavior and ignition times of MB materials.

The time to flameout (TTF) was higher for the hemp (C2) and sawdust (D2) substrate groups that contained the birch bark (BB) additive. The addition of BB also led to increased total heat release (THR) and peak heat release rate (pHRR) values (Table 3). Notably, D2 variant showed significant differences from all other groups in terms of TTF and THR, indicating that including bark reduced the fire performance. This suggests that while BB may enhance other properties of MBs, like reduction in water absorption [14], its presence increases the amount of heat released and prolongs burning, making the material more susceptible to fire hazards. When comparing the M1, M2, and M3 variants, it was observed that the M3 variant had the longest average burning time, while the M1 variant had the shortest. The M1 variant exhibited a statistically significant difference in burning time compared to the M2 and M3 variants. However, no significant differences were found between the M2 and M3 variants. Within the S group, the S1 variant exhibited the longest average flameout time, although this result was not significantly different from that of the S3 variant. In contrast, the S2 variant demonstrated longer average flameout time and a statistically significant difference compared to the other samples.

The total heat release (THR) of MBs was influenced by both the substrate type and composite density. Hemp composites (C group), with half the density of sawdust composites (D group), exhibited nearly three times lower THR. However, the bark additive in the C2 and D2 variants significantly increased the THR to 30 k·W·m^−2^ and 62 k·W·m^−2^, respectively. In contrast, waste fiber composites (M group), despite having higher densities than sanding dust composites (S group), displayed lower THR values (up to 22.3 k·W·m^−2^) than S composites group (55.3 k·W·m^−2^) (Table 3). For comparison, wood composites such as OSB and pine plywood showed much higher THR values, ranging from 98 to 127 k·W·m^−2^ [40,41] demonstrating the superior fire performance of MB materials.

The heat release rate (HRR) is a key factor in assessing fire hazards, as higher HRR values increase the potential for flame spread. The peak heat release rate (pHRR) is particularly important for determining fire performance, as it directly influences building fire safety design. Among the basic MB substrates—hemp (C1), sawdust (D1), waste fibers (M1), and sanding dust (S1)—the lowest pHRR was observed in the M1 variant (134 k·W·m^−2^), followed by C1 (150 k·W·m^−2^), while the highest values were recorded for D1 and S1, at 183 k·W·m^−2^ and 190 k·W·m^−2^, respectively (Table 3; Figure 7). Notably, pHRR values for all MB variants were significantly lower than those of synthetic materials such as XPS and EPS (Table 3). This indicates that mycelium-based materials could offer competitive fire performance, especially compared to wood composites like OSB and pine plywood, which reach higher pHRR values between 240 and 280 k·W·m^−2^ [40,41]. The lower pHRR could be attributed to the formation of the residual char produced by the mycelium. A surface char layer reduces heat release by acting as a thermal insulator and limiting the supply of combustible gases and oxygen to the flame front [39]. The nitrogen presence in the chemical structure of the mycelium promotes the char formation. Nitrogen is structurally and functionally linked with fungal cellular proteins, enzymes, nucleic acids, and chitin [43,44]. The total nitrogen content in the mycelium of *T. versicolor* was reported to be more than 6% [14].

The time to peak heat release rate (TTP) for MB materials varied by variant, ranging from 19 to 31 s. This duration is shorter than that observed for XPS and EPS, which were 33 and 32 s, respectively (Table 3).

Total smoke release (TSR) is a key indicator of smoke hazard and plays a crucial role in evaluating the fire performance of building materials. The TSR for MB variants ranged from 7 to 281 m^2^·m^−2^, with the bark-containing hemp (C2) and sawdust (D2) variants exhibiting TSR values that were 7 to 11 times higher than those of other variants within their respective substrate groups (Table 3). In comparison, rigid polyurethane foam (RPUF) has been reported to have TSR values ranging from 400 to 990 m^2^·m^−2^ [45], while RPUF and polyisocyanurate (PUR-PIR) modified with rapeseed oil-based polyols have TSR values from 204 to 560 m^2^·m^−2^ [46]. Smoke and toxic gases are significant contributors to fatalities and injuries in building fires, a risk that is intensified by the increasing use of synthetic materials and chemical additives in modern construction [47]. In this context, MBs are a promising alternative to synthetic materials, offering probably lower toxicity of released smoke. The chemical composition of the released smoke was not studied as it was out of scope of the present study.

Composites made with waste fibers (M1) exhibited the lowest TTF (149 s), along with the THR (5.8 k·W·m^−2^) and pHRR (134 k·W·m^−2^) among all MB variants, including synthetic foams XPS and EPS (Table 3). WF variants containing co-substrates, such as hemp (M2) or sawdust (M3), demonstrated higher TTF, THR, and pHRR values than M1, indicating that the choice of co-substrate significantly influences the fire properties of the waste fiber composite.

The fire performance of sanding dust (S group) composites was generally comparable to sawdust (D group) composites, with some variations due to specific variants. For instance, the inclusion of a hemp co-substrate (S2) led to reduced TTI, TTF, THR, and pHRR values within the S substrate group. Hemp composites (C group) and those containing hemp co-substrates (M2 and S2) exhibited lower ignition times compared to birch sawdust (D group) and sanding dust (S group) variants. Additionally, THR and pHRR values for hemp-containing composites were lower than those of the sawdust and sanding dust groups, except for variant C2, which included a birch bark additive that increased TTI, THR, and pHRR [42] noted that the flammability properties of natural fibers are generally correlated with their lignin content. Fibers with low lignin and high cellulose content tend to exhibit lower pHRR, attributed to cellulose’s low effective heat of combustion. It leads to charring and incomplete combustion, limiting the contribution of these fibers to the overall heat generated during burning.

To summarize the overall results obtained in this study, some aspects of the potential use of MBs in constructions are noted herewith. The developed thermal insulation is specifically designed for installation between layers of walls, enhancing its effectiveness by utilizing its relatively low thermal conductivity. This characteristic is crucial as it minimizes heat transfer, leading to improved energy efficiency and maintaining comfortable indoor temperatures. To further enhance mechanical performance, MBs can be encased in a rigid shell or reinforced with a bio-based fiber composite. This protective shell not only bolsters the overall durability and strength of the insulation system but also safeguards it from environmental factors. Alternatively, the MB material can be reinforced with a bio-based flax fiber composite. Flax fibers are renowned for their excellent mechanical properties, including high tensile strength and stiffness, which significantly enhance the structural integrity of the assembly. Additionally, being bio-based, they contribute to the sustainability of the overall system. By integrating these materials, it is possible to create a robust and effective insulation solution that addresses both thermal performance and mechanical resilience. This innovative approach harnesses sustainable materials, aligning with contemporary building practices that prioritize environmental responsibility alongside high performance. Even more, MBs are generally considered to be a sustainable and non-toxic materials, offering a safe alternative for indoor use. When working with MBs, cutting or sanding can generate dust that should not pose a greater hazard than the dust produced during the cutting of wood. Once installed, MB materials do not emit harmful volatile organic compounds (VOCs) or mycotoxins. The fungus *T. versicolor* is utilized in its mycelial stage, during which it does not produce or release spores. This species is one of the most studied mushrooms in lignocellulose-based bioeconomy and traditional medicine where it is used in the form of extracts or biomass [48]. Thus, MBs are considered safe for indoor environments and do not pose health risks through ongoing exposure.

## 4. Conclusions

The density of mycelium-based composites was influenced by the primary substrate and the combinations with co-substrates and additives. Including birch bark additive in the hemp and sawdust substrate groups resulted in a higher density. However, the bending strength was more closely related to the combination of substrates than density alone. The highest bending strength was observed in the waste fiber composite with sawdust co-substrate (M3) and in the sanding dust composite with hemp co-substrate (S2), achieving values of 0.45 MPa (ρ = 0.023 g·cm^−3^) and 0.51 MPa (ρ = 0.14 g·cm^−3^), respectively. The bending strength of these MB materials is competitive with that of synthetic polymers like EPS and XPS, and some commercial building materials, such as hempcrete.

The thermal conductivity of MBs was influenced by their density. The lowest conductivity was observed in hemp-based MBs, which had a lambda coefficient of 40 m·W·m^−1^·K^−1^. In terms of thermal properties, hemp MBs can compete with non-mycelium insulation materials, including synthetic polymers like EPS and XPS, as well as glass wool, rock wool, and wood fiber wool.

MBs exhibited superior fire resistance compared to various synthetic foams and composite materials. They demonstrated lower pHRR and TSR than synthetic polymers and lower THR than certain wood composites. The M1 variant, made with waste fibers, had the highest apparent density and exhibited the best fire performance, attributed to the blend of different fibers combined with fungal mycelium. The addition of birch bark (BB) reduced the mechanical and thermal properties, and fire performance. Overall, the mechanical and thermal properties, along with the fire behavior of MBs, support its suitability as a potential alternative to petroleum-derived and traditional composite materials.

## Figures and Tables

**Table 1:** Mycelium biocomposite substrate groups—C (hemp), D (sawdust), M (waste fibers), S (sanding dust) and variants 1, 2, 3. HS = hemp shives; BS = birch sawdust; WF = waste fibers; BSD = birch sanding dust; WB = wheat bran; BB = birch bark.

Variant	Component (g)					
	HS	BS	WF	BSD	WB	BB
C1	600	-	-	-	48	-
C2	420	-	-	-	48	180
C3	648	-	-	-	-	-
D1	-	600	-	-	48	-
D2	-	420	-	-	48	180
D3	-	648	-	-	-	-
M1	-	-	600	-	48	-
M2	300	-	300	-	48	-
M3	-	300	300	-	48	-
S1	-	-	-	600	48	-
S2	300	-	-	300	48	-
S3	-	300	-	300	48	-

**Table 2:** Bending strength (σ_fM_) and elastic modulus (E) results with SD of mycelium biocomposites variants containing hemp (C), sawdust (D), waste fibers (M), and sanding dust (S) as the main substrates; n = 5.

Variant	Density g·cm^−3^	σ_fM_ MPa	E MPa
C1	0.10 ± 0.00	0.11 ± 0.03	5.55 ± 1.26
C2	0.14 ± 0.00	0.15 ± 0.01	5.11 ± 0.47
C3	0.09 ± 0.00	0.10 ± 0.01	4.42 ± 0.27
D1	0.21 ± 0.00	0.25 ± 0.02	14.66 ± 1.76
D2	0.22 ± 0.00	0.17 ± 0.01	8.78 ± 1.31
D3	-	-	-
M1	0.32 ± 0.01	0.39 ± 0.05	26.66 ± 7.34
M2	0.16 ± 0.00	0.42 ± 0.04	9.06 ± 1.98
M3	0.23 ± 0.00	0.45 ± 0.06	9.38 ± 4.46
S1	0.19 ± 0.00	0.10 ± 0.01	4.10 ± 0.17
S2	0.14 ± 0.01	0.51 ± 0.05	17.87 ± 2.88
S3	0.22 ± 0.00	0.32 ± 0.03	5.82 ± 1.92

**Table 3:** Cone calorimeter measurements with SD of mycelium biocomposites variants containing hemp (C), sawdust (D), waste fibers (M), and sanding dust (S) as the main substrates at an irradiance of 35 k·W·m^−2^; n = 4.

Variant	TTI s	TTF s	THR k·W·m^−2^	pHRR k·W·m^−2^	TTP s	TSR m^2^·m^−2^
C1	7 ± 1	372 ± 39	12.5 ± 2.0	150.0 ±0.9	19 ± 1	16.0 ± 3.1
C2	10 ± 0	747 ± 94	30.1 ± 1.5	199.0 ± 2.2	23 ± 1	142.1 ± 7.7
C3	8 ± 1	279 ± 13	12.3 ± 0.5	162.7 ± 6.0	19 ± 1	13.6 ± 0.6
D1	13 ± 2	708 ± 63	33.6 ± 1.7	183.0 ± 10.4	26 ± 3	24.0 ± 4.7
D2	15 ± 1	1181 ± 74	62.1 ± 3.5	181.1 ± 13.4	30 ± 2	281.5 ± 26.2
D3	12 ± 1	711± 12	35.0 ± 2.1	243.1 ± 10.2	25 ± 1	40.6 ± 8.2
M1	15 ± 5	149 ± 69	5.8 ± 0.6	133.9 ± 12.2	26 ± 6	36.0 ± 2.7
M2	9 ± 0	755 ± 21	11.9 ± 5.9	144.6 ± 10.7	21 ± 1	90.7 ± 23.1
M3	18 ± 2	854 ± 61	22.3 ± 4.2	141.5 ± 7.4	31 ± 2	7.4 ± 1.1
S1	14 ± 2	818 ± 363	55.3 ± 6.0	190.2 ± 12.1	23 ± 8	11.3 ± 0.5
S2	9 ± 1	548 ± 25	19.8 ± 2.1	164.2 ± 7.0	21 ± 1	10.5 ± 1.4
S3	13 ± 2	695 ± 57	32.8 ± 5.1	184.8 ± 27.2	26 ± 1	15.3 ± 1.2
XPS [38]	5	174	26.2	423	33	-
EPS [38]	29	135	11.2	377	32	-

## Data Availability

The data presented in this study are available on request from the corresponding author due to ongoing research. The original contributions presented in this study are partly included in the Supplementary Materials.

## Supplementary Materials

The following supporting information can be downloaded at: https://www.mdpi.com/article/10.3390/ma17246111/s1.
