# Supplementary material for: Assessing the Conformity of Mycelium Biocomposites for Ecological Insulation Solutions
Source: Materials (Basel). 2024 Dec 13;17(24):6111. doi: 10.3390/ma17246111 (PMC11677991; doi:10.3390/ma17246111)
Supplement: Supplementary file 1 [file materials-17-06111-s001.zip › Stat summary.pdf]

Statistics

The statistical analysis was conducted using RStudio (R version 4.2.1, "Funny-Looking Kid"). Differences with a p-value < 0.05 were considered significant. ANOVA was employed to assess the significance of differences across groups, followed by Tukey's HSD post-hoc tests for pairwise comparisons. The following R packages were utilized: `agricolae` for performing the ANOVA and Tukey HSD tests, `dplyr` for data manipulation, `readxl` for reading Excel files, and `writexl` for exporting results.

Results

Reaction to Fire

ANOVA revealed significant differences across all tested parameters, including time of ignition, time of flameout, total heat release, peak heat release, and total smoke release ($p < 0.001$).

Post-hoc Tukey HSD Tests

Time of Ignition:
Group C1 and C3 differed significantly from D1, D2, M1, and M3. D2 showed significant differences from M2. C2, D3, M2, S1, S2, and S3 were significantly different from M3.

Time of Flameout:
D2 differed significantly from all other groups. C1 was significantly different except when compared to C3 and S1. M1 was significantly different except when compared to C3. C3 differed significantly from the resto f the groups. M3 was significantly different from S1 and S2.

Total Heat Release (MJ/m^2):
D2 differed significantly from all other groups. C1 and C3 were significantly different from D3. D1 and D3 differed significantly from M1 and M2. M1 and M2 were significantly different from S1.

Peak Heat Release (kW/m^2):
C2 differed significantly from M1, M2, and M3. D3 was significantly different except when compared to C2.

Total Smoke Release (m^2/m^2):
C2 differed significantly from all other groups. D2 and M2 also differed significantly from all others. D3 was significantly different from M3 and S2.

Thermal Properties

ANOVA revealed significant differences in material density and thermal conductivity ($p < 0.001$).

Post-hoc Tukey HSD Tests

Material Density (g/cm³):
C1 differed significantly from D2, D3, M1, S1, and S3. C2 was significantly different from M1 and S1. C3 differed significantly from D1, D2, D3, M1, M3, S1, and S3. S1 was significantly different from M2 and S2.

Thermal Conductivity (mW/m·K):
S1 exhibited significant differences from all other groups.

Bending Properties

D3 was excluded from this analysis due to insufficient replicates.

ANOVA revealed significant differences in material density, bending strength, relative deformation at maximum load, and modulus of bending strength ($p < 0.001$).

Post-hoc Tukey HSD Tests

Material Density (g/cm³):
Significant differences were found between all groups, except between C3 and C1, S2 and C2, M3 and D2, S3 and D2, M3-2 and M2, S1 and D1, and S3 and M3.

Bending Strength (σfM, MPa):
C1 and C3 differed significantly from D1, M1, M2, M3, M3-2, S2, and S3. C2 differed significantly from all groups except C1, C3, S1 and D2. D1 was significantly different from D2, M1, M2, M3, S1, and S2. D2 showed significant differences from M1, M2, M3, M3-2, S2, and S3. M1 differed significantly from M3-2, S1, and S2. M2 differed significantly from M3-2, S1, S2, and S3. M3 was significantly different from M3-2, S1, and S3. M3-2 differed significantly from S1 and S2. S1 was significantly different from S2 and S3. S2 differed significantly from S3.

Relative Deformation at Maximum Load (εfM):
M3-2 differed significantly from all groups except M3 and S2. S2 differed significantly except from C2. S3 was significantly different except from M2 and M3. C1, C2, C3, D1, D2, S1 and M1 were significantly different from M2 and M3. C1 was significantly different from C2. C2 also differed significantly from D1, D2, and M1.

Modulus of Bending Strength (EM, MPa):
M1 differed significantly from all other groups. C1, C2, and C3 were also significantly different from D1 and S2. D1 differed significantly from S1 and S3. Group D2, M2, M3, M3-2, S1, and S3 were significantly different from S2.
