# Supplementary figures and images for: Assessing the Conformity of Mycelium Biocomposites for Ecological Insulation Solutions
Source: Materials (Basel). 2024 Dec 13;17(24):6111. doi: 10.3390/ma17246111 (PMC11677991; doi:10.3390/ma17246111)

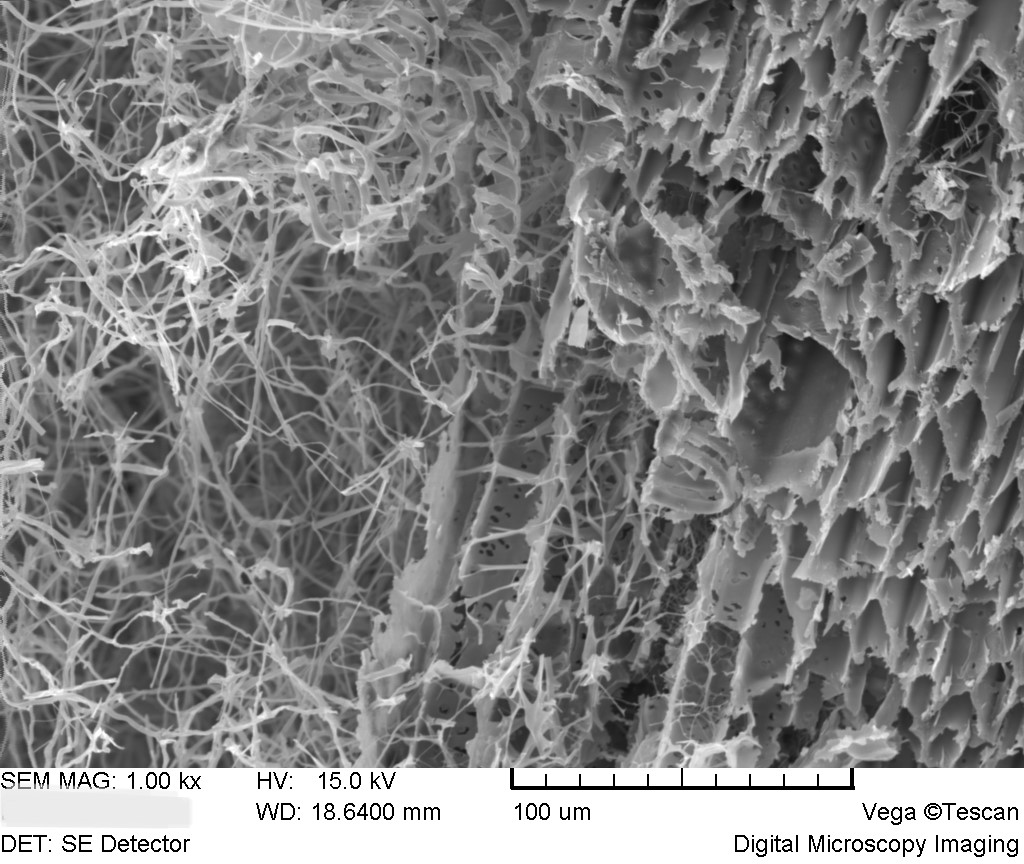

Supplement: Supplementary file 1 [file materials-17-06111-s001.zip › SEM/1000x_C1_1 HS_A.jpg]

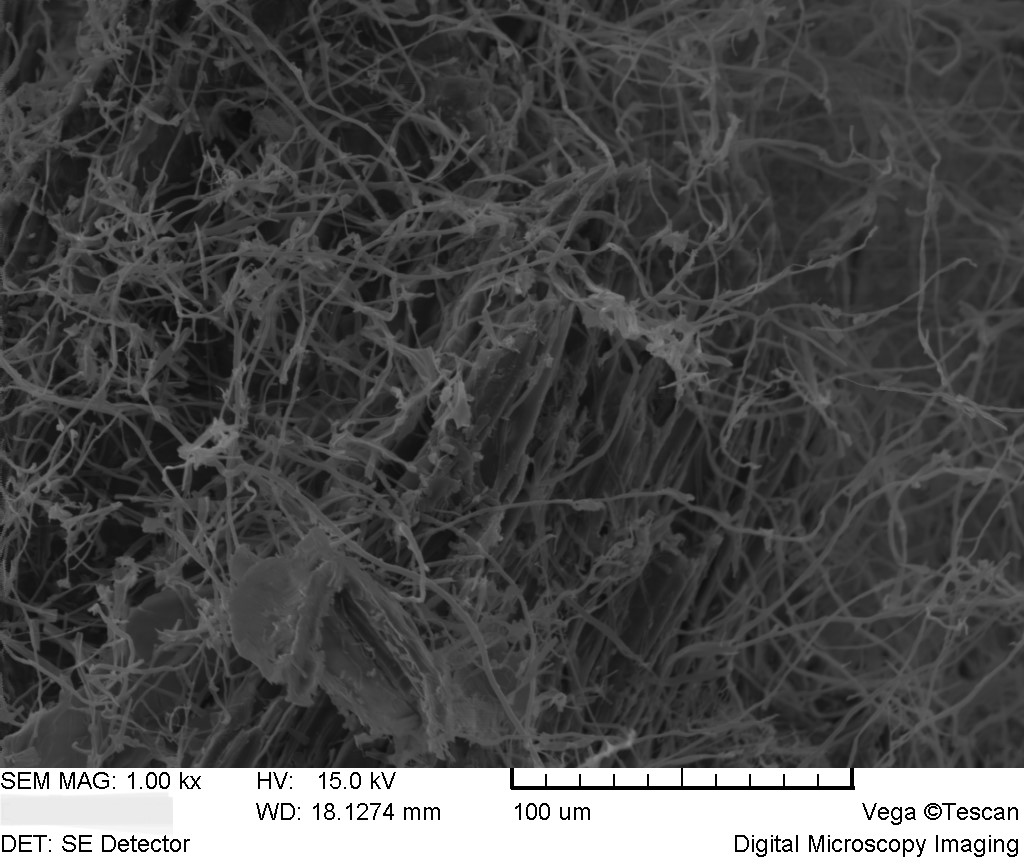

Supplement: Supplementary file 1 [file materials-17-06111-s001.zip › SEM/1000x_D1_5 BS_B.jpg]

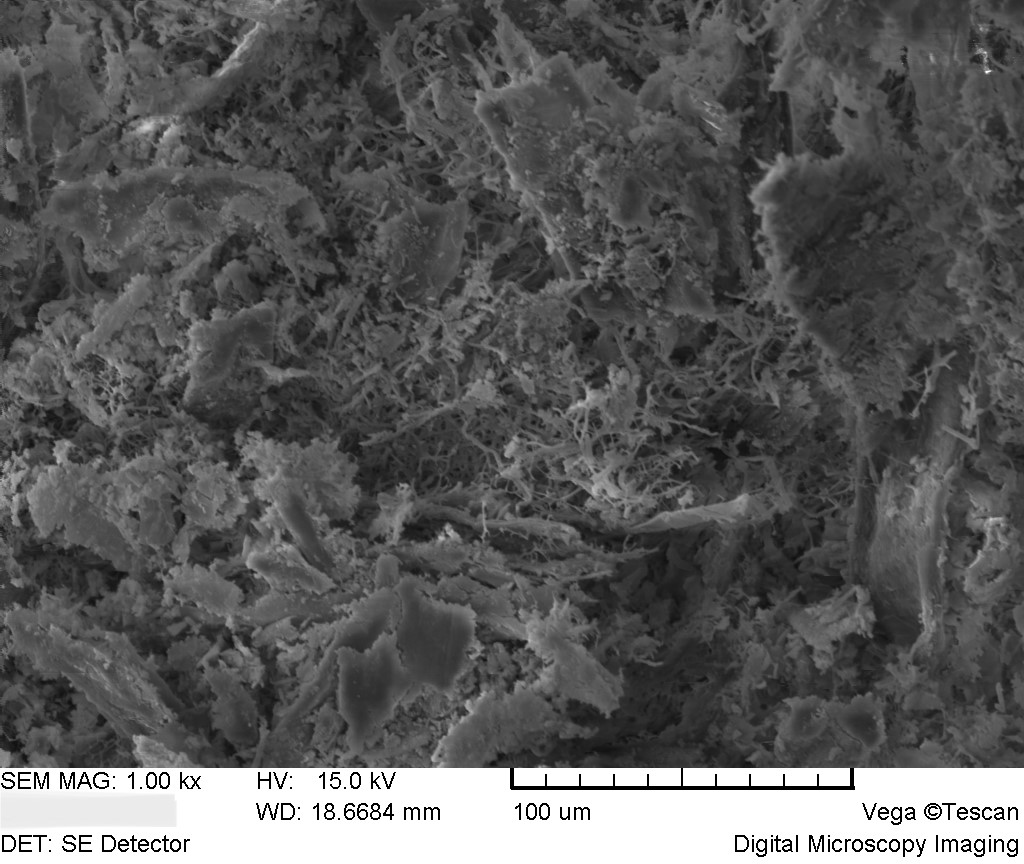

Supplement: Supplementary file 1 [file materials-17-06111-s001.zip › SEM/1000x_M1_4 WF_C.jpg]

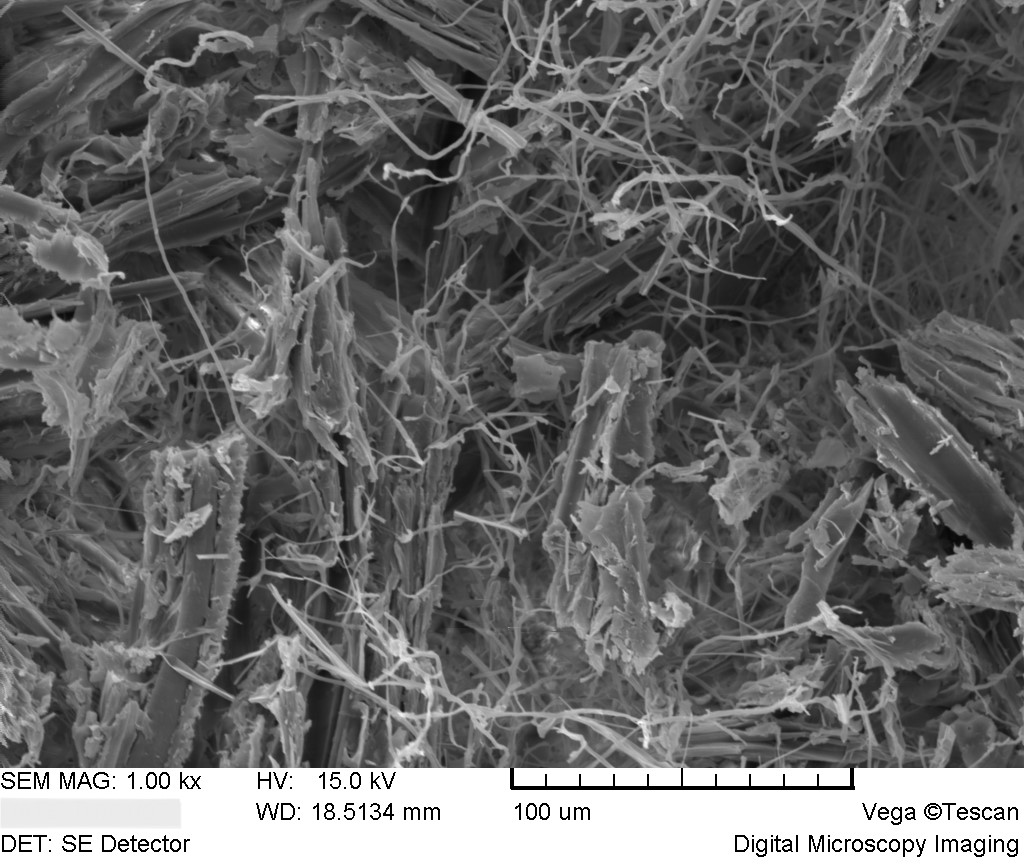

Supplement: Supplementary file 1 [file materials-17-06111-s001.zip › SEM/1000x_S1_1 BSD_D.jpg]

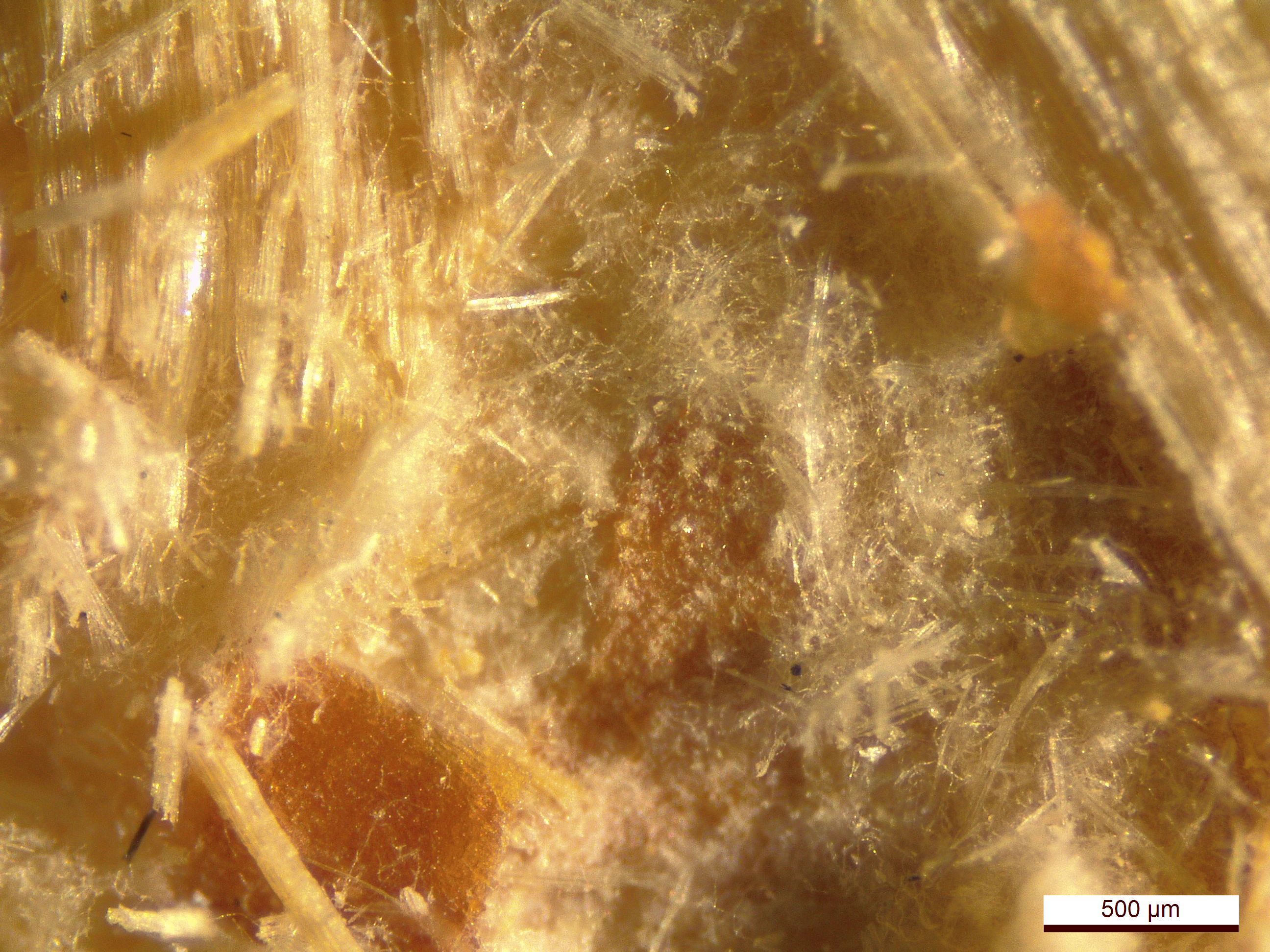

Supplement: Supplementary file 1 [file materials-17-06111-s001.zip › stereomicroscopy/A surf.tif]

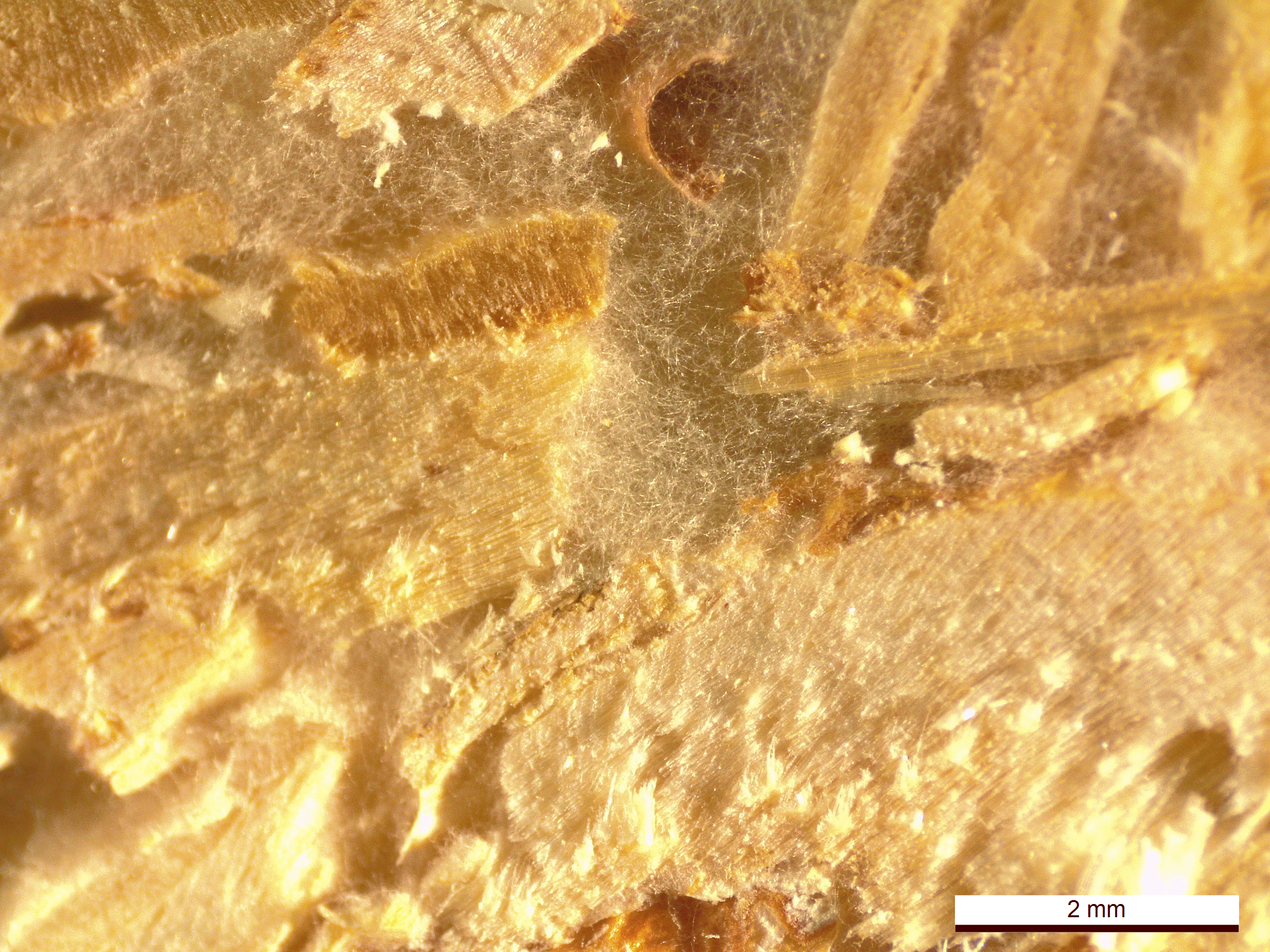

Supplement: Supplementary file 1 [file materials-17-06111-s001.zip › stereomicroscopy/B cross.tif]

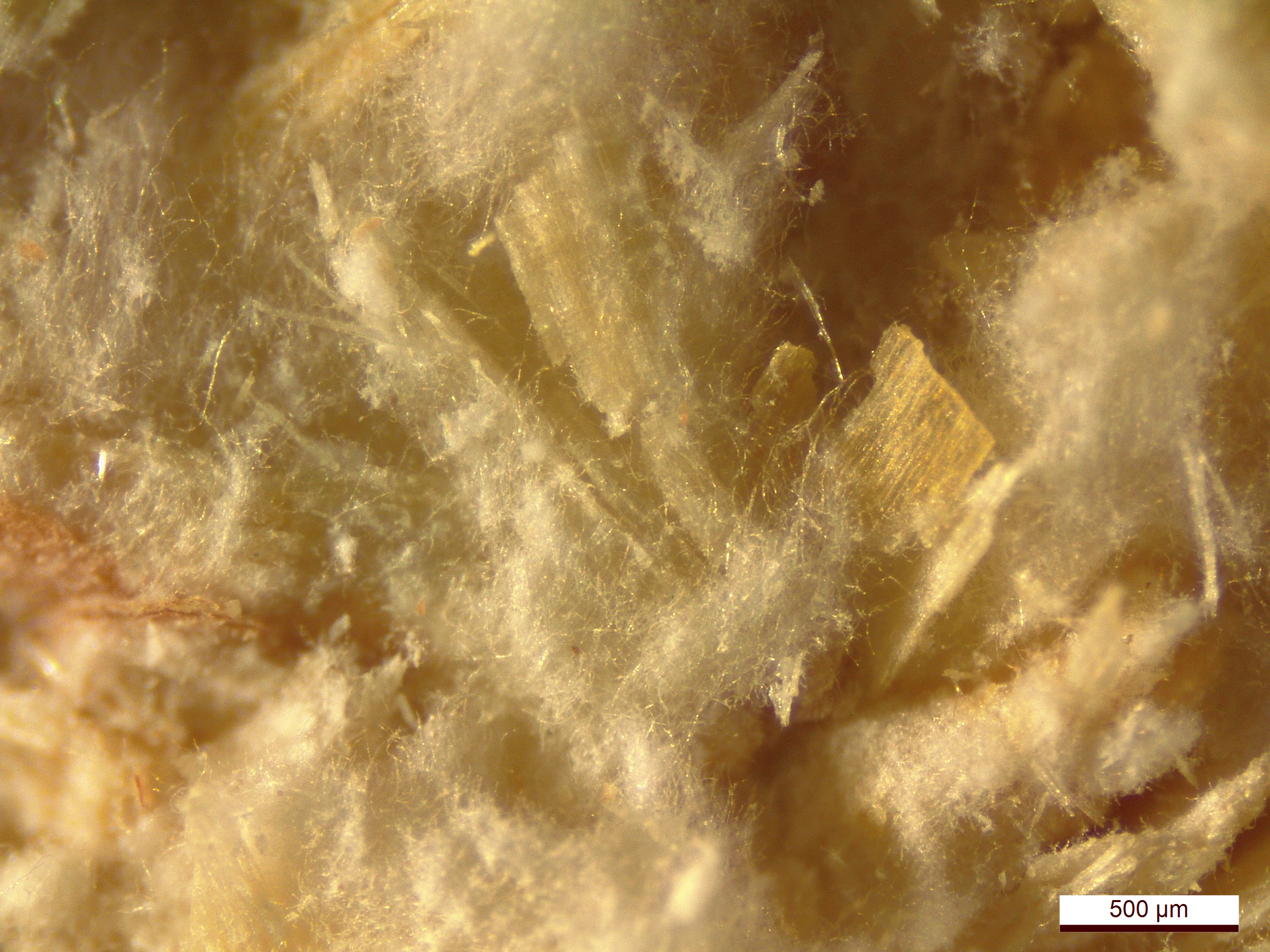

Supplement: Supplementary file 1 [file materials-17-06111-s001.zip › stereomicroscopy/C surf.tif]

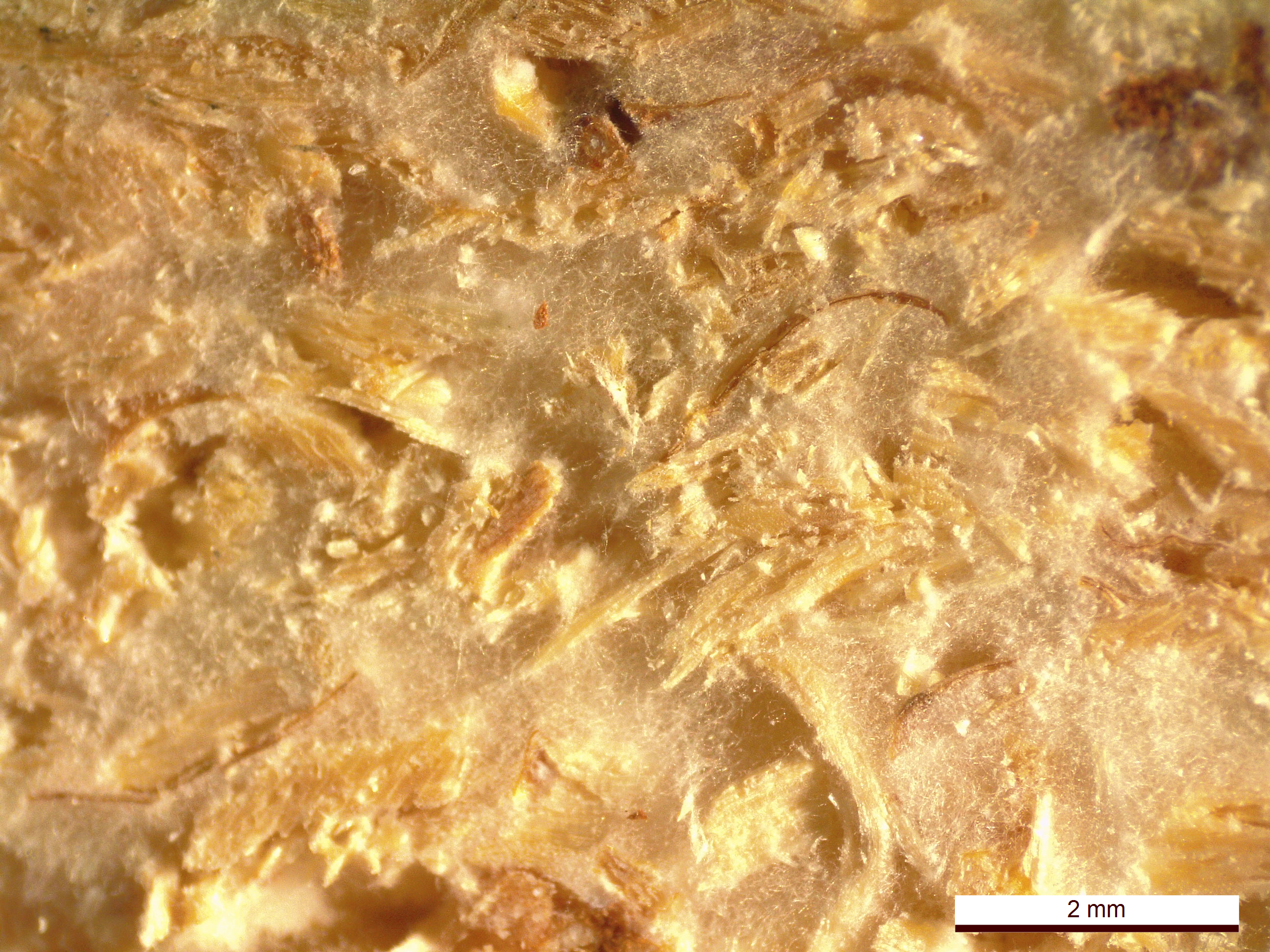

Supplement: Supplementary file 1 [file materials-17-06111-s001.zip › stereomicroscopy/D cros.tif]

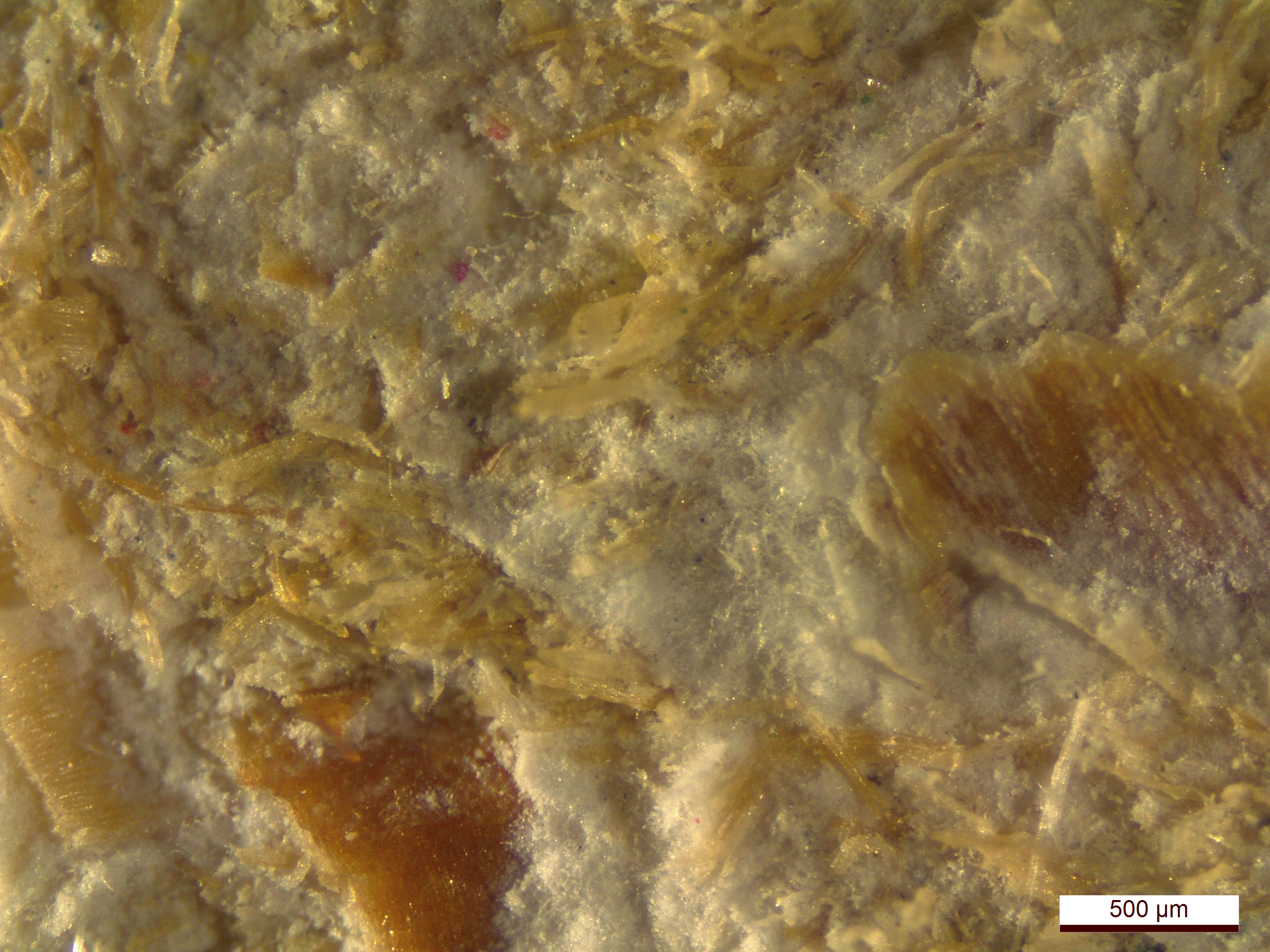

Supplement: Supplementary file 1 [file materials-17-06111-s001.zip › stereomicroscopy/E surf.tif]

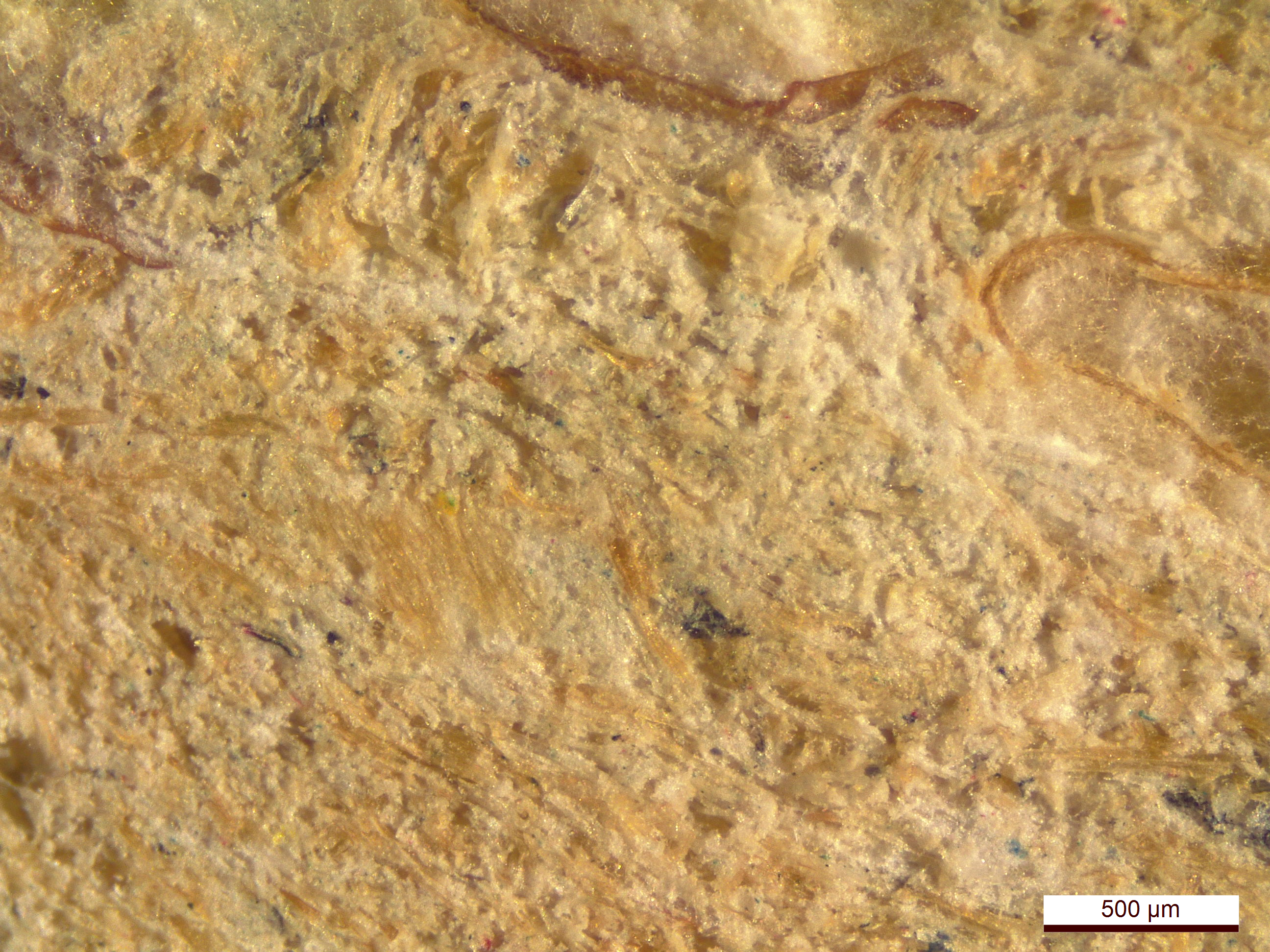

Supplement: Supplementary file 1 [file materials-17-06111-s001.zip › stereomicroscopy/F cros.tif]

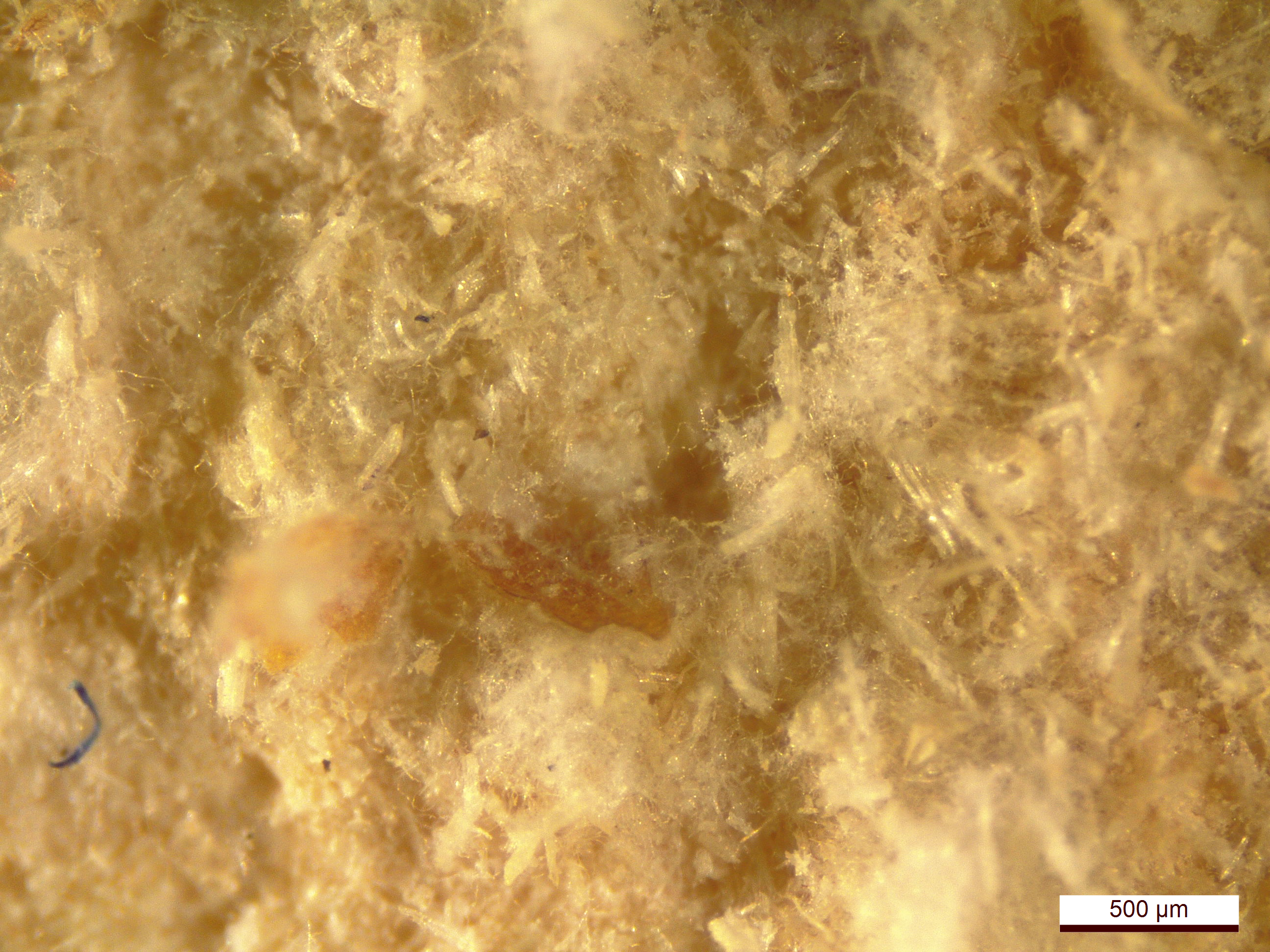

Supplement: Supplementary file 1 [file materials-17-06111-s001.zip › stereomicroscopy/G surf.tif]

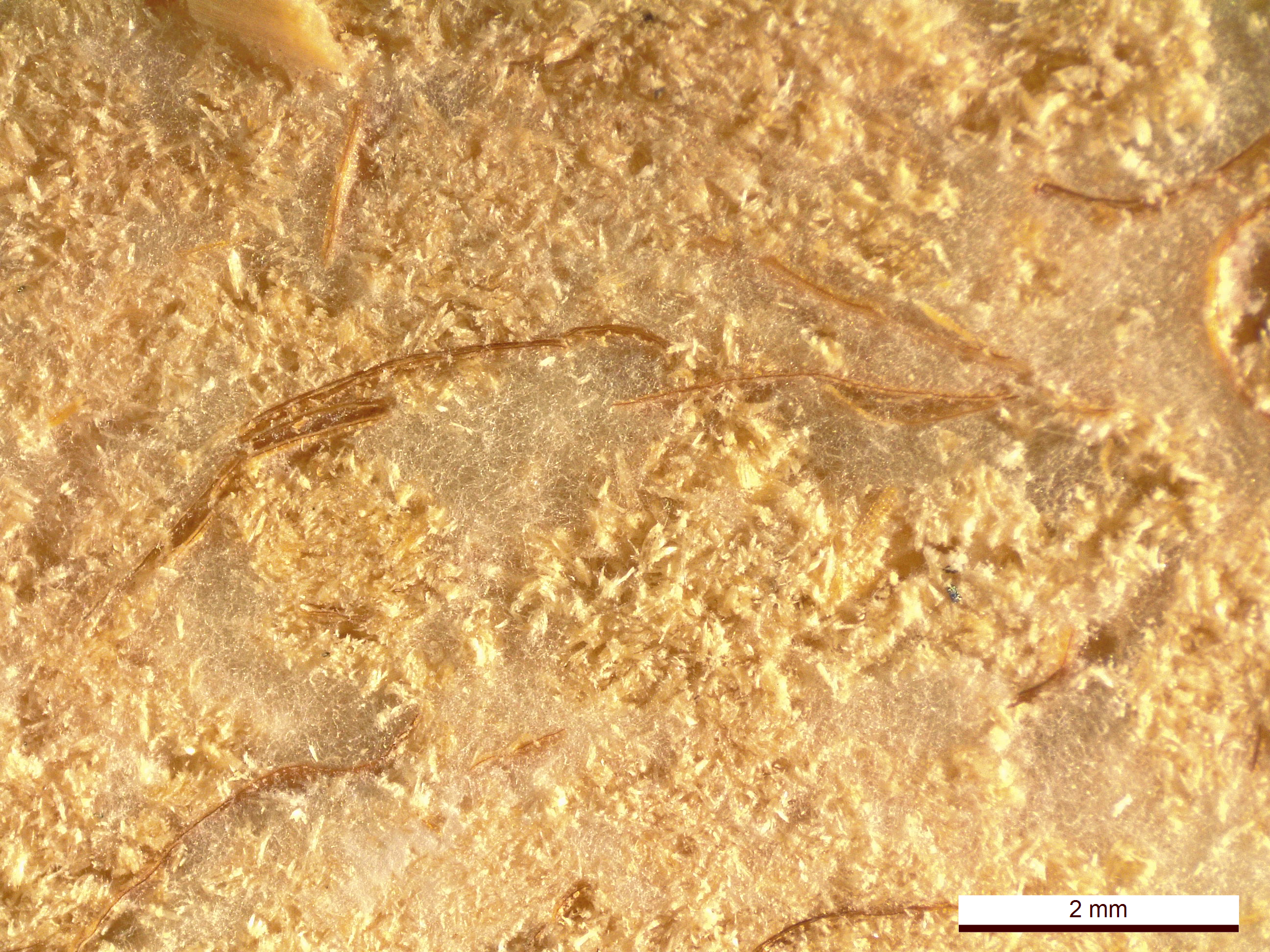

Supplement: Supplementary file 1 [file materials-17-06111-s001.zip › stereomicroscopy/H cros.tif]
